# Outpatients' Perspectives on Problems and Needs Related to Female Genital Mutilation/Cutting: A Qualitative Study from Somaliland

**DOI:** 10.1155/2013/165893

**Published:** 2013-09-10

**Authors:** Sarah Fried, Amina Mahmoud Warsame, Vanja Berggren, Elisabeth Isman, Annika Johansson

**Affiliations:** ^1^Department of Public Health Science, Karolinska Institutet, 171 77 Stockholm, Sweden; ^2^Kristinehamnsgatan 4, 123 44 Farsta, Stockholm, Sweden; ^3^Department of Health Sciences, Faculty of Medicine, Lund University, 221 00 Lund, Sweden

## Abstract

*Aim*. To explore female outpatients' perspectives on problems related to female genital mutilation/cutting (FGM/C) and their views on information, care, and counseling. *Setting*. An FGM/C support center at a maternity clinic in Hargeisa, Somaliland. *Methods*. A qualitative, descriptive study, using content analysis of seven semistructured interviews with female outpatients. *Results*. All participants had been ignorant of the etiology of their FGM/C-related complications and hesitant to seek care. All had undergone infibulation but did not wish the same for their daughters. In recent years they had learnt through religious leaders and media campaigns that infibulation was unapproved by Islam. A less severe FGM/C type, “Sunna,” was more accepted; however, few could define what “Sunna” meant. Condemning and ridiculing attitudes against uncircumcised women prevailed in their community. *Conclusions*. New ideas and concepts related to FGM/C enter the common discourse in the Somali society while traditional norms and values still prevail. Religion was shown to have a strong impact on FGM/C practices and beliefs. Interventions aiming to raise awareness of health consequences of all types of FGM/C, as well as where to seek care for complications, are needed in Somaliland. Involvement of religious leaders in anti-FGM/C programs is essential.

## 1. Introduction 

The term female genital mutilation/cutting (FGM/C) describes procedures of total or partial removal of external female genitalia or other intentional injury to the female genital organs for nonmedical reasons [1]. 

When FGM/C first came to be discussed beyond the societies in which it was traditionally performed, it was generally referred to as “female circumcision”—a term that draws a direct parallel with male circumcision, creating confusion between these two distinct practices [2]. To emphasize the gravity of the FGM/C act, the word “mutilation” was adopted in the 1990s [3]. However, this term can be problematic at a community level, and local languages often instead use the less judgmental “cutting.” In 1999, the UN Special Rapporteur on Traditional Practices drew attention to the risk of “demonizing” certain cultures, religions, and communities. Hence, the term “cutting” has increasingly come to be used [2].

An estimated 100 to 140 million females of the world population have undergone FGM/C. They are most commonly living in east-, west-, and north-east Africa and in some parts of the Middle East and Asia. FGM/C is generally carried out by traditional circumcisers on girls between infancy and 15 years of age [1]. The practice is considered to be a basic violation of a number of recognized human rights. Consequently, it is increasingly addressed and discussed in the context of girls' and women's rights, rather than strictly as a medical issue [1, 3]. 

FGM/C is classified by WHO [4] into four major types (Table 1). Around 90% of FGM/C cases are estimated to include types I, II, or IV, whereas about 10% include type III [3].

FGM/C can cause severe complications [1]. The severity of the injuries is related to the anatomical extent of the cutting, which varies between the different types of FGM/C. Infibulations are associated with additional risks, since they sometimes must be reopened (defibulation) to enable both sexual intercourse and childbirth. This might be followed by reclosure (reinfibulation) and a subsequent need for repeated defibulations. Urinary and menstrual problems can result from the near-complete sealing of the vagina and urethra, causing slow and painful menstruations and urination [5]. 

Being a culturally required and normalized part of girls' upbringing and female adult life, women who have undergone FGM/C often lack knowledge of the normal anatomy and functioning of female genital organs. Thus, they do not associate problems that emanate from FGM/C with this procedure. This ignorance, combined with feelings of embarrassment and shame, may prevent women from seeking medical treatment when needed. This represents a major challenge to programs aimed at alleviating the consequences of FGM/C (Ugaso Lama, NAFIS, personal communication, May 2013). In this paper we describe a project in Somaliland that attempts to target women who have undergone FGM/C with information, counseling, and medical care and to mobilize communities against the practice. 

### 1.1. FGM/C in Somali Culture

Data on FGM/C prevalence in Somalia is scarce, but WHO estimated it to be approximately 98% in 2006. About 90% had undergone infibulation [1]. Traditionally, FGM/C was performed in adolescence as an initiation into womanhood. However, the procedure is no longer considered to be a rite of passage and is today mainly performed on girls aged five to eight. Traditional circumcisers conduct most operations, but the numbers of FGM/Cs performed by professional health providers are increasing [6]. 

Media, religious debates, and anti-FGM/C campaigns by local nongovernmental organizations (NGOs) seem to have activated discussions on FGM/C among the Somali people. This has broken many traditional taboos, resulting in a growing questioning of the practice. People from the Somali Diaspora who return home to Somaliland with more Western views towards FGM/C constitute another important influence. In recent years, a slight shift in FGM/C practice has been observed from infibulation to types I, II, or IV [7].

Somaliland is situated on the eastern horn of Africa and declared independence from Somalia in 1991. Although not internationally recognized, Somaliland has experienced relative stability.

The judicial system in Somaliland still pays little attention to FGM/C, and there is a lack of common policies and coordination of key messages. However, in the National Gender Policy of 2009–2012 [8], FGM/C is highlighted as one of the most flagrant forms of violence against girls and women. In a recent study investigating attitudes toward female circumcision among people in Hargeisa, Somaliland, 97% of the 107 randomly selected female participants were reported to be circumcised. Of these, 81% said they were infibulated [9].

Recently, the Ministry of Labor and Social Affairs (MOLSA) started to monitor and coordinate the development of FGM/C policies. In these activities, the Network Against FGM In Somaliland (NAFIS) is a leading actor from the nongovernmental side.

NAFIS was established in 2006 by 23 NGOs in Somaliland that joined forces to improve the coordination and efficiency of anti-FGM/C efforts in the country. This is made through information, education, advocacy, lobbying, and strengthening of NAFIS member organizations. The long-term objective of NAFIS is a total eradication of FGM/C. Today NAFIS has 19 member organizations. NAFIS works closely with government bodies, media, and religious and community leaders and organizes training of health workers and community members. To meet the need of services for women suffering from the consequences of FGM/C, NAFIS started a support center in 2010 within a maternity clinic in Hargeisa, providing medical care and counseling to these women. By encouraging the patients to advocate for change of the FGM/C practice in their communities and to protect their daughters from the procedure, the center is expected to have a wide impact [10]. Local female volunteers are also recruited from the communities with the task to persuade women with FGM/C-related problems to attend the support center.

In addition to this, NAFIS is running a “community education campaign” in the catchment area of the support center. In recurrent meetings with local women's groups, village health committees, and other local institutions, NAFIS informs the attendees about the negative consequences of FGM/C and where to seek help for FGM/C-related health problems. Community dialogues on FGM/C in the context of religion, human rights, and cultural and social backgrounds are encouraged. It is hoped that NAFIS' messages—total abandonment of infibulation and gradual elimination of all other forms of FGM/C—will be accepted through these ongoing community dialogues. 

## 2. Study Aim

In the spring of 2012, approximately one year after the opening of NAFIS support center, we made a study with the aim to explore the female outpatients' perspectives on FGM/C and related health problems. The specific aims were to explore the women's views on different forms of FGM/C and if/how these views had changed over time; the women's knowledge and own experiences of health consequences related to FGM/C; for what reasons the women had attended the center; who had supported them and what obstacles they had experienced in seeking care; and finally what intentions the women had for their own daughters in relation to FGM/C. In addition, we wanted to investigate the women's perceptions of the care and counseling provided at the support center, and receive their suggestions on how the center could best meet the needs and reach out in their community.

## 3. Study Setting, Material, and Methods 

The study was conducted at a maternity clinic situated in an internally displaced people (IDP) camp in the outskirts of Hargeisa. The area is mainly populated by Somali minority clans subjected to discrimination, and ostracism in the Somali society. This group of clans are collectively known as *Gabooye*, and even though they are mostly skilled people, they are poorer compared to other clans. However, the *Gabooye* share culture, religion and other traditions with the general Somali population, including those related to FGM/C. 

The clinic is one of few health facilities serving these poor communities. It receives outpatients daily and also has a few rooms for deliveries and other inpatients. A gynecologist attends the clinic weekly, and 6–8 nurses/midwives provide maternal care on a daily basis. Within the clinic, the NAFIS support center has one treatment room and one room where women receive counseling in groups or individually by midwives trained by the project. Services at NAFIS support center are free of charge for those who cannot pay.

Exploring experiences of women facing FGM/C-related problems is a delicate issue that requires a sensitive and empathic approach. We chose a qualitative design, using personal interviews to gather information. A semistructured interview guide was prepared (Appendix), including some sociodemographic data and information about the woman's circumcision status, followed by open-ended questions which allowed the interviewee to respond freely and expand on issues of particular interest to her. A trained female Somali social scientist (AW, second author) with experience of qualitative interviewing and a deep knowledge of FGM/C issues in Somaliland performed the interviews in the Somali language.

Study participants were selected by AW among women seeking care at NAFIS Support Center. In agreement with the midwives in charge of the clinic, AW visited the support center throughout three days in March 2012. All the women attending the center during these days, altogether nine were asked if they would be willing to participate in an interview for a research project. The purpose of the study was explained and they were informed that participation was voluntary. Appointments were set up with AW; however, two women failed to attend. They later explained that they did not find the time to talk as they were busy struggling with their daily work (both were street vendors). Thus, altogether seven interviews were conducted. First, one pilot interview was performed, after which some questions were slightly revised for better comprehension. Results from the pilot interview were included in the study. All interviews took place in privacy in the counseling room and lasted for about one hour. They were tape-recorded with the permission of the interviewees, transcribed verbatim, and translated into English by a trained Somali research assistant. The translation was overseen by AW, who is fluent in English, to assure correct comprehension of the translator. Translations of a few Somali words were difficult to find. The research group decided to keep these words in the original language, accompanied by an approximate English translation. 

The collection of data was ceased after the seventh interview, partly due to limitations of time and funding. However, the amount of gathered information was deemed to be sufficient by the research group, as saturation seemed to have been reached and little new information appeared. This was further verified by an experienced midwife working at the support center in examinations of the interviews.

Content analysis was applied to analyze data from the seven interviews (including the pilot interview) [11]. First, the interviews were read and reread several times by the research team members (the five authors). Background characteristics of the interviewees were compiled (Table 2). Five main categories were identified, each one referring to a descriptive level of content and thus expressions of the manifest content of the text. Under each category, a number of subcategories were identified. Two themes emerged from the underlying meaning of the categories [12]. To ensure credibility, the results were discussed and triangulated within the research team, composed of nursing/midwifery and social science disciplines. Representative quotations (from participants identified by numbers in Table 2) from the transcribed text are presented to enhance credibility [12].

The expression “FGM/C” is used throughout this study to capture the significance of the term “mutilation” at policy level and at the same time to recognize the importance of employing, nonjudgmental terminology with practicing communities [13]. For linguistic convenience, however, words such as *circumcise *and *circumcised *are occasionally used.

## 4. Ethical Considerations

All study participants received comprehensive information on the study objectives and methods and were informed that participation was optional. They were promised anonymity and assured that their answers would not affect the health care given at NAFIS support center. All of those who agreed to participate gave their oral consents before participation. Ethical approval was granted by the Ministry of Labor and Social Affairs of Somaliland.

## 5. Findings 

The participating women were born in Hargeisa (3), Berbera (1), or in rural areas (3). Two were unmarried; the others were married or widowed. Three of the respondents belonged to the *Gabooye* minority. The women had sought care for infections, abdominal pain, ruptured uterus, cysts, and/or menstrual problems (Table 2). 

The main categories identified were (1) circumstances inhibiting and facilitating care seeking; (2) views on different types of FGM/C; (3) change of attitudes and practice; (4) social pressure on the uncircumcised; (5) suggestions for the future work of NAFIS (Table 3). Two themes emerging from the categories were seemingly contradictory—on the one hand an apparent change in FGM/C practice and attitudes, and on the other hand the ridiculing attitudes and negative social sanctions that, according to respondents, uncircumcised women face in the Somali society. The major theme running through the interviews was the importance of awareness and access to information. Without this, change in attitudes and behavior related to FGM/C was unachievable. 

## 6. Factors Inhibiting or Facilitating Care Seeking

All participants had been hesitant to seek help for their FGM/C-related health concerns and had suffered for a long time before coming to NAFIS. 

### 6.1. Ignorance, Poverty, and Shame

Lack of information and knowledge, regarding both health consequences of FGM/C and where to find treatment, was mentioned by more than half of the participants as a reason for not seeking care earlier:

*Had I had the knowledge and support from somebody who knew what to do I would have come earlier, but I had neither (Woman 6).*

Other reasons mentioned were poverty and shame:

*I had been hiding my problem from everybody for quite a long time. I was ashamed to tell anybody. It was my secret … I was not married when the cyst appeared. Later, I became too sick and was unable to walk, yet still ashamed until I decided to seek help … If I knew this was related to FGM/C and that there were other women suffering like me and that there was help, I would have sought care (Woman 3).*

*… ignorance, poor economy, and taking things easy and ignoring your problems until it becomes worse. In my case I did not have the money to seek care (Woman 1).*

Some had been hiding their problems for many years. One woman stated:

*Out there, there are many sick women who are hiding their problem. Some even die due to their problem… (Woman 7).*

### 6.2. Ease of Access and Social Support

Some of the participating women had received information about the support center since they were living in the vicinity. A few were referred to NAFIS by other health facilities, while others had been encouraged to seek help by female friends, neighbors, or relatives who had either received help from the support center themselves, or knew someone who had.

Of the four participants living with their husbands, two were accompanied by their husbands when they visited the support center. Another wanted her husband to come with her, but since he was away at the time, she brought a female friend instead. One woman brought her mother, and another one brought a female neighbor. Two participants came by themselves. 

## 7. Views on Different Types of FGM/C

The participants clearly differentiated between what they referred to as “Pharaonic circumcision” (infibulation/FGM/C type III) and “sunna circumcision” (FGM/C types I, II, or IV). There was a broad consensus against infibulation, whereas most respondents had a positive attitude towards sunna—although they were unable to clearly explain what sunna was.

### 7.1. Infibulation

All of the women participating in the study were infibulated (i.e., had undergone the most severe type of FGM/C). However, none of them could see any advantages of infibulation. They all agreed that infibulation brings about several health problems:

*I do not see any advantage with Pharaonic circumcision. If the nature of the genitals are changed it is a problem. Think of a door of a house is completely shut. Is this not a problem? (Woman 1).*

*I do not see any advantages with Pharaonic circumcision … Your genitals are sliced and stitched and the menses is blocked and very painful. All kinds of ills follow (Woman 2).*

*It [infibulation] has no benefits but is a curse for women (Woman 3).*

### 7.2. Sunna

All of the participants were firmly convinced of the negative health effects of infibulation. However, a common notion was that no health problems derive from less severe forms of circumcision. Sunna was seen as a safe alternative to infibulation, preferred for both physical and religious reasons:

*When I was circumcised in the old way, most people thought it was good and I did not see any problems myself. But today things have changed and people are doing the sunna type. Sunna is better and has no health related problems and our religion has told us to do it … I changed my mind on the Pharaonic type around 10 years ago … I realized it was a bad tradition and un-Islamic and after I heard from religious leaders the sunna is better and should be followed. There is also a general trend for people to abandon the Pharaonic type and change to sunna (Woman 4).*

One participant stated that sunna circumcision is necessary to remain healthy. Her answer to the question “do you think, healthwise, there is a problem if a girl is untouched?” was

* Yes, as she is with the baaro (A derogatory word connoting that the clitoris is filthy or dirty) and it needs to be bled (Woman 4).*

Another woman had learnt that sunna must be practiced according to religious law, but she could not explain what sunna circumcision means:

*I do not have an idea about it as I did not see anybody … but I heard that infibulations has [sic] been abandoned nowadays and girls are circumcised on sunna (Woman 2).*

One participant pointed out that there was not much difference between sunna-cut girls and “untouched” girls:

*…there is not much difference between the untouched and the sunna circumcised girls since neither of them is stitched. Only a small cut at the top is the difference between those who are not touched and those who had the sunna (Woman 7).*

However, another woman had a different definition of what sunna means:

*I would advise that girls should be spared from circumcision of any type because even in the sunna type people still use stitches (Woman 3).*

A third woman described the sunna circumcision that had been performed on both her daughters:

*They cut a little part of the clitoris and made one stitch (Woman 7).*

### 7.3. Plan for Own Daughters

Only two of the participating women had decided to leave their daughters “untouched.” One of them explained that she made this decision because she did not want her future daughters to undergo the same suffering as she did. She stated:

*I do not expect I will ever circumcise my daughter. I do not care if she can find a husband or not. I do not want her to have my experience (Woman 3).*

Four out of the seven interviewees had decided to perform, or had already performed, sunna circumcision on their daughters. One woman had not made up her mind yet:

*I plan to do a slight sunna, or I can even leave them [the daughters] as they are (Woman 1).*

Of the five participants who were married, four said that their husbands would not oppose the decision they had made regarding their daughters' circumcision.

## 8. Change of Attitudes and Practice

Our findings pointed at changes in FGM/C practice from Pharaonic to sunna circumcision. This shift in both attitude and practice was said to be influenced by both religious leaders and information through media. 

### 8.1. Religious Concerns and Influences

Several of the participating women stated that they started opposing Pharaonic circumcision when they learnt that the practice was un-Islamic. However, these women had heard from religious leaders that sunna is approved by the Islamic law:

*It has no benefits whatsoever, besides God does not allow it. When I had the menstrual pains and I was told that the problem was caused by FGM/C, my first doubts started. After I listened to the radio and saw the TV programs about FGM/C, my views also changed. I also learned that religion does not allow FGM/C … Religion approves of the sunna type (Woman 7).*

*Even before the awareness raising, I heard that FGM is not religious. Later I found out on the many problems. I think the majority of the people are aware now that it is not religious … Sheiks say sunna must be practiced but religion prohibits the Pharaonic … When circumcising her daughter every mother asks the sheiks, who advise them to circumcise on sunna (Woman 2).*

Others had received contradictious messages regarding sunna, saying either that it has to be done according to Islam, that it should be antagonized, or that it is optional:

*I heard advocacy for sunna but not for total abandonment (Woman 6).*

*I heard over the radio some religious scholars saying that it should be stopped and others saying sunna should be applied (Woman 3).*

Seemingly, the women had received unclear messages from religious leaders regarding FGM/C. None of the participants had heard a local sheik or religious scholar promote Pharaonic circumcision, nor had anyone of them heard advocacy for total abandonment of the FGM/C practice. The third alternative, sunna, was either presented as a religious prescription, or more vaguely as something optional, left for each individual to relate to. This picture of unclear and sometimes contradictive messages described by the participating women was confirmed by the midwives working at the support center. 

### 8.2. Turning Points in Change of Attitude and Increased Openness

Both own and others' FGM/C-related physical problems were mentioned as strong underlying motives for a shift in attitude towards FGM/C by nearly all participants. Some also referred to increased awareness through media campaigns. One woman stated that she had started opposing the practice when she came to NAFIS and learned that her dysmenorrhea derived from FGM/C:

*When the doctor opened me, I got relieved of my menstruation problems. From that day on, I'm against FGM/C (Woman 6).*

*[I started to oppose FGM/C] when this girl at NAFIS told me that the cause of my problems and pain with menstruation is due to my genitals being cut and closed and that this is against religion. (Woman 6).*

One participant had previously been recommended by a doctor to undergo defibulation due to her abdominal problems, but she refused to undergo the operation:

*I used to have very painful menses when I was a girl and before I got married I used to vomit. The doctor suggested that I be opened, but I did not do it because it was shameful to be opened those days (Woman 7). *

All participants said that they nowadays often discuss FGM/C issues with female friends, relatives, and neighbors. None of them perceived any difficulties in discussing the subject, even though one woman said she was a little shy to discuss it with men. Several of the participating women expressed positive attitudes towards the increased openness in their society; one stated that she thinks all people should take part in the discussions; another one said that she wants everyone to know about the problems that FGM/C brings about. One woman emphasized that she would like to include men in the discussions:

* … I hear men say, look at these women talking about their private parts in TV or radio. When they say this I say to these men, do not feel surprised about this as women are suffering from FGM/C and it is also good for you men that FGM/C is abandoned (Woman 1).*

Another woman added that she even heard that fathers discuss the FGM/C status of their daughters with their future son-in-laws:

*I heard that nowadays when a girl is getting married, her father tells the husband to be that his daughter is circumcised in the sunna type lest that the husband becomes surprised when he sees her open (Woman 7).*

## 9. Social Pressure on the Uncircumcised

The social sanctions and consequences that a woman who had not undergone FGM/C would face in her community were vividly discussed in the interviews. 

### 9.1. Bullying

According to the interviewees, people would talk and call an uncircumcised woman names, referring to her “openness” and implying that she was not a virgin:

* Though it's not like before, yet people will say bad things about a girl who is untouched. They will say things like “she is open” or that she is not a virgin (Woman 3).*

*She will be called bad names and people will say she is with the baaro or she is buuryo qab (“She still has the clitoris”) (Woman 4).*

*The woman neighbors who were told [would] always laugh and wonder how their genitals would [look] like without any sort of cutting. Many see it as unnatural to be left alone (Woman 6).*

The words that are used to describe an uncircumcised woman, “with the *baaro,*” are associated with something dirty and filthy. The biologically normal untouched female genital with intact clitoris is described as unnatural, strange, and ugly. By definition an untouched woman does not belong to the range of “normal” women. Moreover, she is suspected of immorality. 

### 9.2. Reduced Marriageability

Breaking social norms, and being “unnatural” and “different” from other girlsg were reported as problems for uncircumcised women. Above all, being uncircumcised was said to have a negative impact on a girl's chances of getting married. All participants agreed that an uncircumcised woman might have problems finding a husband:

*Such a girl may meet insults if she is known not to be circumcised and that will have an impact on her future marriage. Some men might not like to marry an uncircumcised girl (Woman 5).*

However, others were less pessimistic:

*I do think she will find somebody to marry. People are with the times and they are not like before (Woman 4).*

## 10. Knowledge about NAFIS and Suggestions for Future Work 

Asked about knowledge of NAFIS' work and policies, one participating woman responded that NAFIS thinks that all forms of FGM/C should be stopped and also gives training. Four participants said that NAFIS raises awareness and provides counseling and seminars where they strongly advice against Pharaonic circumcision. One of them stated:

*NAFIS raises awareness against Pharaonic circumcision. They also hold seminars against it. When NAFIS gives us the awareness raising, we also pass it on to other women that the sunna is ok but Pharaonic circumcision is bad and should be stopped (Woman 4).*

One participant had a more imprecise understanding of NAFIS' work: “I only heard that they [NAFIS] help people with problems …” (Woman 2).

Almost all participants stated that they were content with the care they received at NAFIS Support Center. Some added that they now felt better and that their health had improved. However, one participant had continuing problems with painful menstruations and infections. A couple of women mentioned that they were encouraged during their visit to tell other women about NAFIS and their messages.

Asked for suggestions on how NAFIS' and similar support centers' work could be improved, all participants emphasized the importance of raising awareness among women, especially in poor and rural areas. One wished for more seminars and education, while others stressed that women should be informed and encouraged to seek help immediately and not wait until their problems are deteriorated. 

Media campaigns were said to be a good way for NAFIS to reach out. One woman requested more training “especially to women, as women are a key to stopping the practice” (Woman 1). Another one suggested that education and training should focus on both mothers and fathers, and also on practitioners. More information to rural communities was suggested, since “they are the ones who have the least information and they are the ones who suffer most” (Woman 5). One participant pointed out that most women who need support are poor and cannot afford to seek help, and consequently NAFIS should inform people that they give treatment for free.

## 11. Discussion 

Strong disapproval of infibulation came out as a major finding in the interviews, although all of the women participating in the study had undergone infibulation themselves. None of them would let their daughters go through the same procedure; however, the less severe FGM/C type called “sunna” was accepted as an alternative. All participants in our study agreed that infibulation brings about severe health problems, while all but one considered sunna to be free of risks.

The trend of a shift in FGM/C practice from type III to types I, II, or IV (“sunna”) has been described in several previous studies performed in other Eastern African countries, such as Sudan [9, 13, 14], and among Somali refugees in Ethiopia [15]. The view of sunna as an innocuous procedure corresponds with previous findings from Somaliland [7, 16] and Sudan [14]. 

In our study, the participating women claimed to be in favor of sunna but could not explain what sunna means and had never seen a sunna-circumcised woman. Similarly, previous research has shown that the term sunna is used loosely in Somaliland to describe a variety of degrees of cutting and that mothers often leave the decision on how much to cut to the practitioners [7]. 

The participants believed that Islamic law approves of sunna; however, they had received contradicting messages from religious authorities. In previous studies, religious concerns are frequently mentioned as an important reason for the shift in FGM/C practice. While infibulation is said to be against Islam, sunna circumcision is thought to represent a milder form of FGM/C, dictated by the Quran [13–16]. Missailidis and Gebre-Medhin [17] have shown that infibulations decreased in Eastern Ethiopia in favor of sunna after a campaign by Muslim leaders. According to Warsame and Talle [7], many religious leaders in Somaliland regard anti-FGM/C campaigns to be part of a “Western agenda,” propagated by international NGOs through local women's organizations. Instead they advocate sunna, claiming that this procedure distinguishes Muslim women from Western women. Few religious leaders in Somaliland, or other parts of eastern Africa, seem to take side against all types of FGM/C. Yet, their large impact on people's behavior might be a key to abandonment of the FGM/C practice. Involvement of Islamic leaders has been reported to be an essential strategy for change of FGM/C behavior in Muslim communities [18]. 

Another major finding in our study was the feelings of shame and fear of being put to scorn if not undergoing any type of FGM/C. Somali Bantu women in the US have been reported to perceive FGM/C as a way to remain “clean” by Upvall et al. [19]. Other authors who have discussed cleanliness and purity as reasons for the continuation of FGM/C are Dirie and Lindmark [20], Gruenbaum [21], and Van der Kwaak [22]. Exposed to name-calling and gossiping, uncircumcised women are still considered to be “unclean,” and the untouched female genitals to be “filthy” or “dirty.” The clitoris is described as an unclean, shameful, and unwanted part of the female body. The statement “I believe that a girl should be bled … The *baaro* needs to be bled” (Woman 4) indicates that for some people, total abolishment of FGM/C is still not an option—they can accept that infibulation is being abandoned, but still some kind of cutting has to be performed on girls' clitorises. 

The subject of negative social sanctions affecting uncircumcised women has been addressed in a number of previous studies. Results from Senegambia have shown how uncircumcised women face harassment, insults, and ostracism from other circumcised women [23]. According to Gruenbaum [21], Sudanese parents who neglect the responsibility to protect their daughter's virginity by FGM/C are criticized by the society, subjecting the family to dishonor. However, in our study, some participants stated that getting married would not be impossible for an uncircumcised woman as long as she is still a virgin. The comment “people are with the time and they are not like before” (Woman 4) suggests that a change of discourse may be underway in the society. The statement one participant made regarding her daughter: “I do not care if she can find a husband or not. I do not want her to have my experience” (Woman 3), suggests that for this woman, being unmarried is no longer associated with the same stigma as it once was. A mother stating that she does not care about her future daughters' marriageability may indicate that parents start to see other options in life than just marriage for their daughters.

None of the participants saw any difficulties in discussing the subject of FGM/C. On the contrary, they stated that they wished for even more openness with both men and women participating in the debate. These findings correspond with what previously has been shown; media, religious debates, and campaigns seem to have broken many traditional taboos and actuated discussions on FGM/C in Somaliland, resulting in a trend of doubting its value [7].

The most significant theme emerging from the interviews was how increased knowledge of the harmful effects of infibulation had profoundly influenced the participating women's change of attitude. On the personal side, they had their own experiences of long-term pain and gynecological problems. Realizing that these problems derived from FGM/C was an eye-opener to them. The information received through media campaigns and awareness programs had resulted in a more open social climate where women could speak out about and share experiences of the consequences of infibulations. What had been regarded as “normal” for a woman in traditional culture was now being questioned. Being “open” was no longer as shameful. 

Participants' suggestions about the future work of anti-FGM/C support centers like NAFIS stressed the needs to continue to raise awareness by media involvement and improved outreach. Breaking the ignorance, embarrassment, and shame that prevent women from seeking care and treatment is the major challenge for NAFIS and similar programs aiming to alleviate the consequences of FGM/C.

## 12. A Note on Moral Authority in Research on FGM/C

As pointed out previously by Sala and Manara [24], people from cultures practicing FGM/C might regard disapproval by other societies as misguided or as cultural imperialism. A common objection expressed by practitioners is that outsiders should not pass moral judgments on other people's rituals. Shell-Duncan and Hernlund [25] state that a central question for all scholars involved in efforts to eradicate FGM/C is *who (if anyone) has the moral authority to condemn the FGM/C practice?* Globally, there is an ongoing debate between cultural relativists and universalists. The doctrine of cultural relativism holds that no culture has the moral authority to criticize another; each act can, according to social approval or disapproval, be considered as good or bad. The universalist standpoint, on the other hand, is that certain values are the same everywhere, independently of cultural differences [24]. FGM/C violates a number of human rights, such as the rights to nondiscrimination, to integrity of the person, to the highest attainable standard of health, and to freedom for children from violence and maltreatment [26]. Balancing between the relativist and universalist standpoints with regard to FGM/C as a cultural phenomenon, we recognize that achieving sustainable change of the FGM/C practice in the Somali society, aiming at total eradication, requires great sensitivity and respectfulness towards the people whose lives have been deeply affected by the practice.

## 13. Study Limitations and Suggestions for Further Research

This qualitative interview study was performed with seven female outpatients visiting the support center. The women who accepted to be interviewed were all against infibulation; they saw a trend of change from infibulation to sunna, and they all stated that they felt free to discuss FGM/C-related problems. Due to limitation of time and budget we could not increase the number of interviews. On the other hand, when seven interviews had been conducted, little new information seemed to be coming forth. In addition, saturation was confirmed by one of the experienced midwives at the center after being presented to the content of the interviews. Nevertheless, the question of potential bias is important to consider. Of the nine women asked to be interviewed, two opted out claiming they were very busy. We cannot rule out that they actually did not want to talk about their problems. Furthermore, these women might have been poorer than the ones who accepted to participate and might also have been less exposed to NAFIS' information and community outreach. Thus, the women interviewed might have been more positive to NAFIS' messages than those who declined. However, according to the midwives/counselors working at the center, the seven women interviewed seemed representative of most outpatients visiting the center.

In qualitative studies there are no claims to generalize to a larger population; it is the depth of understanding and the trustworthiness of the findings that are important. Do the findings truly reflect the views and attitudes of these women in relation to the topics discussed? We firmly believe that they do. The interviewer (AW) holds deep knowledge of the socio-cultural context of FGM/C in Somaliland. She communicated daily with the midwives/counselors at the clinic and could discuss and compare her impressions of the stories told by the women with them. 

The findings raise important questions for further research. One major finding of great concern is why women seem to accept “sunna” and claim it to be free of risks, while at the same time they cannot describe what it involves. It would be valuable to conduct further research in different areas and among different groups to explore the changing understanding of and meaning assigned to sunna for the various stakeholders. A few previous studies have been done, for example, in 2010 by Warsame and Talle [7] on the concept and meaning of sunna among Somalis in Hargeisa. Their results show that the term sunna often is used loosely, referring to various degrees of cutting. 

A broader research area is the social patterning of knowledge, perceptions, and practices regarding the different types of FGM/C between urban/rural populations and educational and socioeconomic groups, as well as between minority groups. In-depth qualitative studies, combined with data from clinic registers on the frequency and complications of various forms of FGM/C in the areas concerned, could elucidate changing practices. They could also be used in the planning and priorities of women's health care. The situation of the nomad women is especially urgent, since these women live far away from health facilities and without access to media information. 

The role of men in the decision making regarding circumcision of daughters, as well as in supporting their wives to seek care, was briefly mentioned by some of the interviewees but not probed into in depth. However, as the main decision makers in the family and society, the involvement of Somali men is obviously of great importance in supporting their wives and in turning the tide towards abandonment of FGM/C. For example, one study participant mentioned that nowadays when a girl is getting married, it is common that her father informs the future husband that his daughter is circumcised in the sunna type. Young Somali men's views on FGM/C, and whether they expect their future wives to have undergone the procedure, are important leads for further investigations.

## 14. Conclusions 

The findings of this study concur with trends observed in previous research of an ongoing change in FGM/C practice in Somaliland. They indicate a process of modernization in the Somali society where new ideas and concepts related to FGM/C enter the common discourse, changing attitudes and practices and questioning the FGM/C custom. At the same time traditional social norms and values still prevail, and “un-touched” women are frowned upon and regarded as “abnormal.” The study illustrates how the power of culture, which defines normality and forms of female identity, takes its toll when the “untouched” women are bullied and ridiculed.

Religion has a strong influence on beliefs and practices of FGM/C. This observation concurs with recent literature from Somaliland. Dialogue with and involvement of religious leaders seem to be of great importance for further FGM/C education and awareness campaigns. Particularly, their position concerning sunna is a crucial issue to clarify. 

Local anti-FGM/C centers offering care and counseling to women, combined with mobilization work at community level, seem to raise awareness, increase willingness to abandon infibulation, and support women to seek help for FGM/C-related problems and issues. Further education and campaigns to raise awareness are probably needed in Somaliland to inform the citizens about the negative health consequences of all types of FGM/C and where to seek care for FGM/C-related problems. Moreover, additional research on the ongoing processes of change in knowledge, attitudes, and practices related to FGM/C in the Somali society would provide necessary further data. Additional information on these areas is essential for the development of policies aiming at abolishing FGM/C and alleviating its consequences for girls and women.

## Figures and Tables

**Table 1:** Classifications of FGM/C according to WHO [4].

Type I, clitoridectomy	Total or partial removal of the clitoris and/or the prepuce.
Type II, excision	Total or partial removal of the clitoris and the labia minora, with or without excision of the labia majora.
Type III, infibulation	Narrowing of the vaginal opening through creation of a covering seal by cutting and repositioning of the inner or outer labia, with or without excision of the clitoris.
Type IV, other	Includes all other harmful procedures to the female genitalia for nonmedical purposes, such as pricking, piercing, scraping, incising, or cauterization.

**Table 2:** Background characteristics of participants.

	Woman 1	Woman 2	Woman 3	Woman 4	Woman 5	Woman 6	Woman 7
Age	35	23	35	30	20	23	40
Origin	Rural area	Rural area	Hargeisa	Hargeisa	Hargeisa	Berbera	Hargeisa
Education/job	Sales woman	No education, no job	No education, no job		No education, no job	No education, but is literate. no job	Quranic school, no job
Marital status	Married	Married	Widowed and remarried	Married	Unmarried	Unmarried	Widowed
Type of FGM/C	Pharaonic	Pharaonic	Pharaonic	Pharaonic	Pharaonic	Pharaonic	Pharaonic
Number and sex of children	1 son, 2 daughters	2 sons	1 daughter	1 daughter, 2 sons	—	—	2 sons, 2 daughters
Problems from FGM/C and reasons for visiting NAFIS	*“… during the process of giving birth, the place between my anus and vagina tore open (fistula).” *Also had infections.	*“I was pregnant and the fetus died in my womb. This happened with 9 babies … I [also] have pain in my abdomen all the time*.”	*“I came to the centre in search of information on a cyst that I had ‘under' here … I became too sick and was unable to walk …” *	*“I had an infection. When I was a girl, before marriage, I used to have very painful menses.” *	*“I had a painful infection … I had menstrual problems and pain in abdo-men.” *	*“I used to have very painful menstruations. My periods did not come out normally as the opening was so narrow.” *	*“I had pain in my abdomen. I used to have very painful menses … and I used to vomit.” *

**Table 3:** Data analysis.

Main categories	Subcategories		
Factors inhibiting and facilitating care seeking	Lack of knowledge	Poverty and shame	Ease of access, social support
Views on different types of FGM/C	Infibulation	Sunna	Plan for own daughters
Change of practice and attitudes	Religious concerns and influences	Turning points	Increased openness
Social pressure on the uncircumcised	Bullying	Reduced marriage-ability	
Knowledge about NAFIS and suggestions for future work	Misunderstandings	Positive attitude	Raising awareness, outreach

## Appendix

### Interview Guidelines

The overall aim is to explore from the women's perspective the quality of care and counseling given at the NAFIS Support Center.

1. Background information
  - Age?
  - Live in urban/rural area. Internal refugee?
  - Number of years of education? Work?
  - Married?
  - Number of children, number of daughters?
2. FGM/C-related health problems and care
  - Why did you visit the NAFIS Support Center?
  - Did you seek care immediately when your problems emerged, or have you had these problems for a long time?
  - *If the respondent waited to seek care*: Why did you wait? What could have made you seek care earlier?
  - What type of FGM/C have you undergone (pharaonic, other)?
  - Have you had any other problems related to FGM/C? What kinds of problems?
  - What kind of help have you got during your visit at NAFIS Support Center? Medical care? Counseling?
  - Did your husband participate in your visit at NAFIS Support Center?
  - *If no*: Would you have wanted him to come?
  - *If yes*: Who decided that he would come (you, him, both of you, other family member)?
  - Did any other member of your family participate in your visit? Who?
  - From whom did you first hear about NAFIS?
  - Are you content with the care given to you at the center? Why/why not?
  - Do you have any suggestions on how the care at NAFIS Support Center could be improved?
  - Would you recommend other women to seek care at NAFIS?
3. Views on advantages/disadvantages of FGM/C
  - Do you see any advantages with FGM/C? Do you see any disadvantages? Why/why not?
  - *If the respondent cannot see any advantages*: When did you come to be against FGM/C? What made you come to this conclusion?
  - Is there a difference in health consequences depending on type of FGM/C (pharaonic, other)?
  - Do you think sunna circumcision is more acceptable from a religious point of view?
  - Do you think a girl who has not undergone any FGM/C would face problems?
  - *If yes*: what kinds of problems (e.g., social problems, marital problems, health problems)?
4. The home context
  - With whom (if anyone) do you usually discuss matters of FGM/C, FGM/C-related problems, and so forth?
  - Do you find it hard to talk about FGM/C? Why/why not?
  - Have your daughters undergone FGM/C/do you intend to circumcise your daughters? Why/why not?
  - *If yes*: what kind of FGM/C did you/do you plan to perform on your daughters?
  - Do you discuss this matter (FGM/C of daughters) with your husband? Do the two of you agree on the matter? Why/why not?
  - Are there any other member(s) of your family who has an influence on the decision of circumcising or not circumcising your daughters?

(5) NAFIS
  - What do you know about NAFIS and about the network's policy on FGM/C?
  - Do you have any ideas on more ways for NAFIS to reach out with their anti-FGM/C message in your family/community?
